# Mixed *Mycobacterium tuberculosis* Lineage Infection in 2 Elephants, Nepal

**DOI:** 10.3201/eid2505.181898

**Published:** 2019-05

**Authors:** Sarad Paudel, Chie Nakajima, Susan K. Mikota, Kamal P. Gairhe, Bhagwan Maharjan, Suraj Subedi, Ajay Poudel, Mariko Sashika, Michito Shimozuru, Yasuhiko Suzuki, Toshio Tsubota

**Affiliations:** Hokkaido University, Sapporo, Japan (S. Paudel, C. Nakajima, M. Sashika, M. Shimozuru, Y. Suzuki, T. Tsubota);; Elephant Care International, Hohenwald, Tennessee, USA (S.K. Mikota);; Department of National Parks and Wildlife Conservation, Kathmandu, Nepal (K.P. Gairhe);; German Nepal Tuberculosis Project, Kathmandu (B. Maharjan);; National Trust for Nature Conservation, Lalitpur, Nepal (S. Subedi);; Chitwan Medical College, Chitwan, Nepal (A. Poudel)

**Keywords:** elephants, tuberculosis, lineage, tuberculosis and other mycobacteria, Mycobacterium tuberculosis, TB, Nepal, bacteria, zoonoses

## Abstract

Tuberculosis in elephants is primarily caused by *Mycobacterium tuberculosis*. We identified mixed *M. tuberculosis* lineage infection in 2 captive elephants in Nepal by using spoligotyping and large sequence polymorphism. One elephant was infected with Indo-Oceanic and East African–Indian (CAS-Delhi) lineages; the other was infected with Indo-Oceanic and East Asian (Beijing) lineages.

*Mycobacterium tuberculosis* is a primary cause of tuberculosis (TB) in elephants (*1*). Culture of trunk wash samples is regarded as the standard method for the diagnosis of TB in elephants; however, this method has many limitations (*2*). We previously reported TB in 3 elephants in Nepal that was caused by *M. tuberculosis* of Indo-Oceanic lineage (*3*). Here, we report on mixed *M. tuberculosis* lineage infection in 2 captive elephants from Chitwan National Park (CNP) in Nepal.

Elephant A was a female elephant ≈65–70 years old. She had been in retirement for 3 years before she died in February 2013. We observed TB-like lesions in the lungs postmortem (Appendix Figure 1). Elephant B was a 32-year-old male. His body condition had substantially deteriorated for several months before he died. We found extensive TB-like lesions in the lungs at postmortem. 

We performed the DPP VetTB Assay (Chembio Inc., http://chembio.com), a serologic test, on the postmortem lung fluid (an off-label use) of elephant A and the serum of elephant B; results were reactive in both cases, indicating the presence of antibodies to TB. We processed the suspected lung lesions according to standard guidelines (*4*) and performed culture by using Löwenstein–Jensen media.

We performed genetic analyses on the 2 *M. tuberculosis* isolates by using spoligotyping and large-sequence polymorphism (LSP) as described previously (*5*). We amplified the direct-repeat region with a primer pair and hybridized the PCR products to a set of 43 oligonucleotide probes corresponding to each spacer covalently bound to the membrane. We identified the spoligo-international type by comparing spoligotypes with the international spoligotyping database (SpolDB4) (*6*). We performed LSP on the isolates by using specific primers for respective lineages, as described previously (*7*).

We identified the elephant isolates as a mixture of 2 strains based on uneven spoligotyping color development (suggesting mixture) and LSP detection PCR results (2 bands were observed). The spoligotyping results showed that the elephant A isolate had a new spoligotype that was not found in the international spoligotyping database. The elephant B isolate belonged to the Indo-Oceanic lineage (East African–Indian 5 spoligo-international type 1365) (Table). The prevalence of the Indo-Oceanic lineage among human TB patients in Nepal is only 11.5% (*8*). The drug resistance–associated region sequences *rpoB*, *katG*, *inhA* promoter region, and *gyrA* were all wild types in both isolates. Similarly, LSP results showed that elephant A was infected by the Indo-Oceanic and East African–Indian lineages (CAS-Delhi) (Appendix Figure 2), whereas elephant B was infected with the East Asian type (Beijing type) (Appendix Figure 3). The prevalence of CAS-Delhi and Beijing type lineages in Nepal in human TB patients is 40.6% and 32.2%, respectively (*8*). In the *gyrA* sequence, both of the samples showed a mixed peak of T231C, suggesting that the East African–Indian type is a Nepal-specific lineage. 

**Table:** Genotypic characteristics of *Mycobacterium tuberculosis* isolates from 2 elephants, Nepal*

Source	Spoligotype† binary code	SIT	Clade	*gyrA*‡
Elephant A	1110000111111111111111001111000010111111111	New§	New§	T231C
Elephant B	0000000000000000000000001111000010111111111	1365	EAI5	T231C

*EAI5, East African–Indian 5; SIT, spoligo-international type. †Spoligotype was determined as previously described by Brudey et al. (*6*).  ‡Mutation in a partial sequence of *gyrA*. The *gyrA* sequence of both elephant isolates had a synonymous single nucleotide polymorphism from T to C at position 231. §Not found in the international spoligotyping database (SpolDB4).

Our study shows that the first elephant was infected with the Indo-Oceanic and East African–Indian (CAS-Delhi) *M. tuberculosis* lineages, whereas the second elephant was infected with the Indo-Oceanic and East Asian (Beijing) lineages. We previously identified the Indo-Oceanic lineage in 3 elephants from Nepal (*3*). We suspect that this lineage might be well adapted in elephants in Nepal.

We diagnosed the mixed lineage infection postmortem in both elephants. However, a successful antemortem diagnosis of mixed infection in a single elephant would enable a precise TB diagnosis and selection of an appropriate anti-TB treatment, which could eventually lead to the control of this disease at the herd level.

The source of these mixed infections is unknown and could be from humans or elephants infected with these lineages. Infected elephant handlers who have daily close contact would be a likely human source. Genotyping of additional isolates from elephants and their handlers will help to determine the source of infection. We recommend regular TB screening of elephant handlers to safeguard human health and help prevent transmission of TB from humans to elephants.

Appendix Additional information regarding mixed *Mycobacterium tuberculosis* lineage infection in 2 elephants, Nepal.
