# Supplementary material for: Mixed Mycobacterium tuberculosis Lineage Infection in 2 Elephants, Nepal
Source: Emerg Infect Dis. 2019 May;25(5):1031–2. doi: 10.3201/eid2505.181898 (PMC6478232; doi:10.3201/eid2505.181898)
Supplement: Appendix — Additional information regarding mixed Mycobacterium tuberculosis lineage infection in 2 elephants, Nepal. [file 18-1898-Techapp-s1.pdf]

Article DOI: https://doi.org/10.3201/eid2505.181898

Mixed *Mycobacterium tuberculosis* Lineage Infection in 2 Elephants, Nepal

Appendix

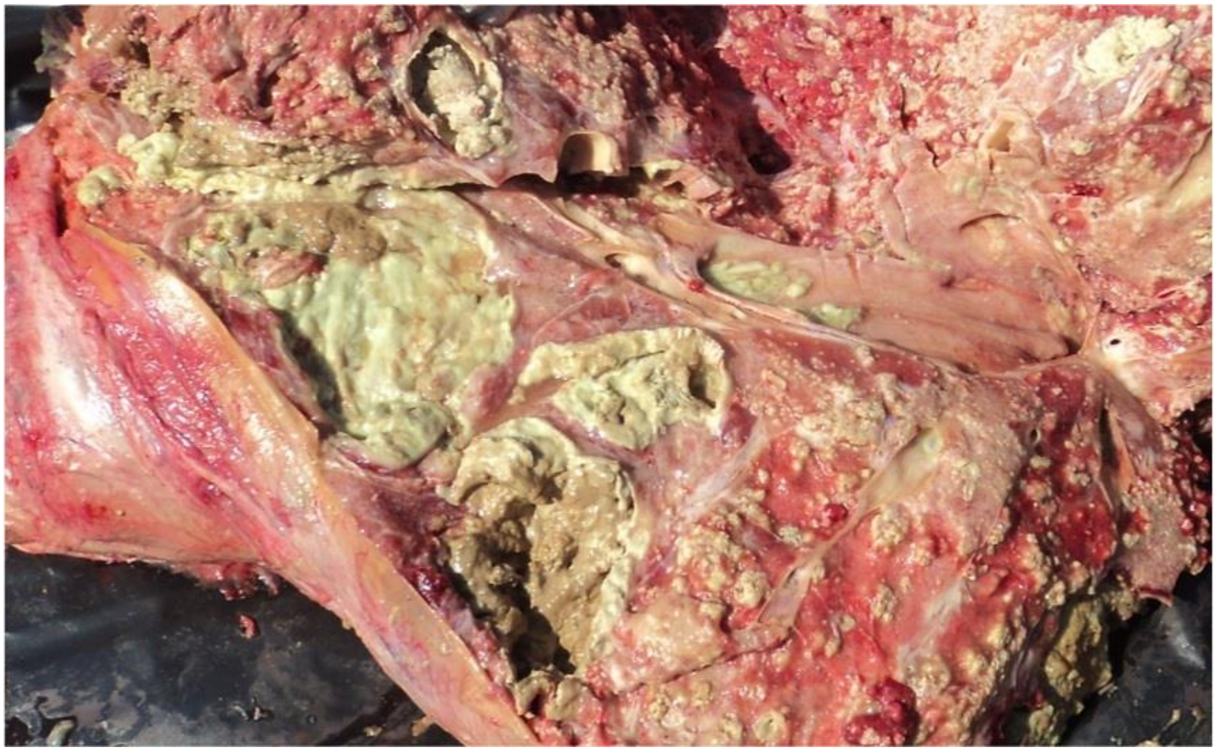

Appendix Figure 1. Granulomatous tuberculosis lesion with caseous mass in lungs of Elephant A.

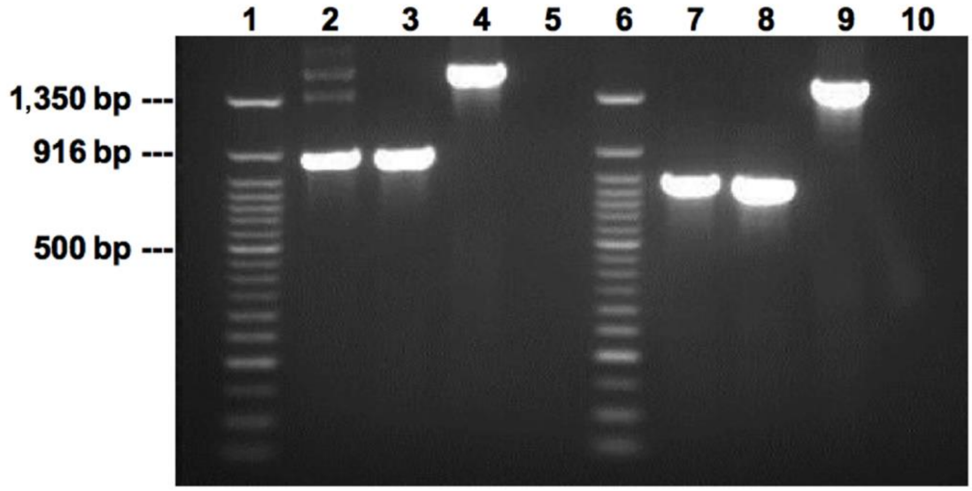

Appendix Figure 2. Large Sequence Polymorphism (LSP) results of Elephant A isolate. Gel electrophoresis was run in isolate A using primers for Indo-oceanic lineage (lineage 1) and East-African-Indian lineage (lineage 3). Lanes: 1, 50bp DNA ladder; 2, Elephant A isolate; 3, positive control for Indo-Oceanic lineage; 4, H37Rv (Euro-American lineage, lineage 4); 5, negative control; 6, 50bp DNA ladder; 7, elephant sample; 8, positive control for East-African-Indian lineage; 9, H37Rv; 10, negative control.

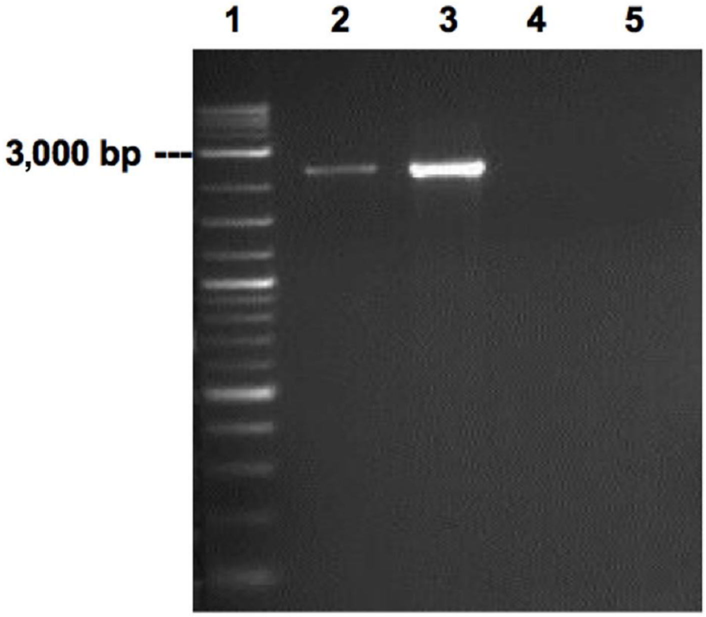

Appendix Figure 3. Large Sequence Polymorphism (LSP) results of Elephant B isolate. Gel electrophoresis was run in isolate B using primers for East-Asian-Beijing (lineage 2). Lanes: 1, 2-log DNA ladder; 2, Elephant B isolate; 3, positive control for East-Asian-Beijing; 4, BCG; 5, negative control.
